# Explaining public satisfaction with health‐care systems: findings from a nationwide survey in China

**DOI:** 10.1111/hex.12429

**Published:** 2015-11-23

**Authors:** Neil Munro, Jane Duckett

**Affiliations:** ^1^ School of Social and Political SciencesUniversity of GlasgowGlasgowUK

**Keywords:** China, health systems, performance, public perceptions, satisfaction

## Abstract

**Objective:**

To identify factors associated with health‐care system satisfaction in China.

**Context:**

Recent research suggests that socio‐demographic characteristics, self‐reported health, income and insurance, ideological beliefs, health‐care utilization, media use and perceptions of services may affect health‐care system satisfaction, but the relative importance of these factors is poorly understood. New data from China offer the opportunity to test theories about the sources of health‐care system satisfaction.

**Design:**

Stratified nationwide survey sample analysed using multilevel logistic regression. Setting and participants: 3680 Chinese adults residing in family dwellings between 1 November 2012 and 17 January 2013.

**Main outcome measure:**

Satisfaction with the way the health‐care system in China is run.

**Results:**

We find only weak associations between satisfaction and socio‐demographic characteristics, self‐reported health and income. We do, however, find that satisfaction is strongly associated with having insurance and belief in personal responsibility for meeting health‐care costs. We also find it is negatively associated with utilization, social media use, perceptions of access as unequal and perceptions of service providers as unethical.

**Conclusions:**

To improve satisfaction, Chinese policymakers – and their counterparts in countries with similar health‐care system characteristics – should improve insurance coverage and the quality of health services, and tackle unethical medical practices.

## Introduction

This article makes a theoretical and empirical contribution to understanding the factors associated with public satisfaction with health‐care systems. Public satisfaction is important because members of the public are beneficiaries and actors in health systems, and their opinions can be important in shaping health policies, providing feedback on the quality and responsiveness of services, and in bringing legitimacy and accountability to the policymaking process.^1^, ^2^, ^3^, ^4^ Unlike patient satisfaction studies, public satisfaction research includes non‐users as well as users of health care, and incorporates not only experiences based on service provision but also wider factors – such as ideological beliefs and media influences.^5^, ^6^ But public satisfaction research remains undertheorized because there have been few attempts at synthesizing the results of prior research and very few data sets include the variables needed to test existing hypotheses rigorously.

Although theory is underdeveloped, previous research does point to some likely key influences. The most commonly reported are socio‐demographic characteristics, which are thought to influence satisfaction by shaping people's expectations.^7^, ^8^, ^9^ In former Soviet countries, for example, rural residents are more satisfied with health‐care systems.^8^ In Europe and Israel, older people are more satisfied.^7^, ^9^, ^10^, ^11^ Similarly, in Europe, Israel and the former Soviet Union, those with less education tend to be more satisfied,^8^, ^9^, ^10^ though one study in Western Europe found that those with least and with most education are more satisfied than those with a middle level of education.^7^ In each of these examples, rather than concluding that those who are more satisfied enjoy better services, researchers interpret socio‐demographic effects as reflecting differing expectations. Finally, although most studies find gender is not significant,^7^, ^8^, ^10^, ^11^ Missinne *et al*.^9^ found that women in Western Europe are less satisfied with health‐care systems than men, but they did not offer any explanation.

Some studies have considered the effects on satisfaction of self‐reported health, finding that people who report poor health tend be less satisfied than those who report good health.^7^, ^9^, ^11^ There are different explanations of why this should be the case: some researchers argue that poor health shapes people's experiences of the health‐care system, especially in less well developed systems.^11^ There is also some evidence that satisfaction is lower among people who report poor mental health.^7^, ^8^

Other studies suggest that satisfaction with health‐care systems is reduced when people encounter financial barriers to access. Donelan *et al*.^12^ found, for example, that in the United States, where a substantial proportion of the population does not have health insurance, uninsured citizens were less satisfied with the health‐care system than their insured counterparts. In addition, Blendon *et al*.^13^ found that Americans with below average incomes were less satisfied than those with above‐average incomes. Missinne *et al*.^9^ meanwhile found that in Western European countries, where insurance is relatively comprehensive, people with low incomes were *not* generally less satisfied with their health‐care systems. Others have shown that in the former Soviet Union and in Israel, where the insured often need to make significant co‐payments, those who are less satisfied with their household economic situation tend to be less satisfied with health‐care systems.^8^, ^10^

The relationships between satisfaction and financial barriers to access are not always clear, however. A five country survey in 2001 showed that in Canada (where health care is free at the point of use) and New Zealand (where people make only modest co‐payments), people with below average incomes were still significantly less satisfied than those with above‐average incomes.^13^ This may be because even low co‐payments are unaffordable for some, because income influences the standard of care people receive, or because people on low incomes have poor access to care because of where they live.^13^, ^14^

There is some evidence from Western Europe that health‐care system satisfaction is affected by people's ideological beliefs. Employing a generic measure of ‘egalitarianism’ based on two questions about the fairness and acceptability of differences in income and living standards, Missinne *et al*.^9^ found people holding egalitarian welfare values to be less satisfied with their health‐care systems than those not holding those values. This finding is surprising as Western European health‐care systems are among those that provide most comprehensively and equitably for their citizens. How values affect health‐care system satisfaction outside of Europe is an open question.

Some studies indicate that health‐care system satisfaction is affected by utilization, implying that direct experience of health‐care providers changes people's opinions. In a study of 21 European countries, people who had used health‐care services in the last 5 years were found to be more satisfied than those who had not, although the effect varied by country.^7^ By contrast, in the former Soviet Union, non‐users were found to be more satisfied in every country except Russia.^8^ A positive utilization effect suggests the health‐care system is better than reputed among the population, while a negative utilization effect suggests it is worse.

Researchers have speculated that satisfaction may be affected by media use: those who pay attention to mass media reporting of the health‐care system may be influenced by its positive or negative portrayals.^7^, ^8^ Such speculation may be founded in awareness of the differences in satisfaction between users and non‐users. Those who have used the health‐care system may base their opinions on experience, but non‐users may be much more reliant on the media, so that inaccurate portrayals could explain why users and non‐users have different opinions. So far, however, there has been little empirical evidence to support such claims.

Finally, some studies have argued that health system satisfaction is influenced by perceptions of health‐care services.^12^, ^15^ There is as yet, however, no clear picture of the dimensions of services that matter. In Europe, Bleich *et al*.^7^ found satisfaction to be associated with people's perceptions of their autonomy and choice, as well as provider communication, respect for dignity, prompt attention and the quality of basic amenities, while Wendt *et al*.^11^ found satisfaction to be associated with perceptions that doctors spend enough time with patients. It is likely that different factors play a role in different health‐care settings.

This article reports on an analysis of the factors associated with health‐care system satisfaction in the research discussed above. It does so using data from a stratified nationwide random sample survey carried out in China in late 2012 and early 2013. The survey was specifically designed to examine theories about popular evaluations of health‐care systems. The richness of the data set allows simultaneous testing of all the major explanations of public satisfaction with health‐care systems.

From systems of communally provided health care, China moved during the 1980s and 1990s to a fee‐for‐service system in which most health‐care costs were borne by patients and their families.^16^ The value of employer‐provided health‐care benefits eroded and in rural areas co‐operative health‐care systems collapsed. Beginning in the late 1990s, the government moved to gradually rebuild social insurance systems, of which there are now three main types, Urban Employees Basic Medical Insurance, Urban Residents Basic Medical insurance (for the non‐employed), and so‐called New Rural Cooperative Medical Schemes (for those with agricultural residence registration including most migrants). By the time our survey fieldwork began in late 2012, more than 90% of Chinese citizens belonged to one or another of these three types of schemes, giving access to reimbursement for health‐care costs amounting to between 40% and 70% of inpatient costs with a ceiling of six times the average wage for city employees or six times average disposable income for non‐employed urban residents and farmers.^17^

The survey took place almost 3 years after the Chinese government in 2009 announced major health system reforms. These reforms aimed at achieving comprehensive basic health‐care coverage by 2020 and addressing a number of serious problems, including high out‐of‐pocket payments, inequitable access, overcrowding of hospitals, and misaligned incentive structures that encouraged medical professionals to generate revenues from certain services and medicines.^16^, ^17^, ^18^, ^19^ Despite gradual expansions of insurance coverage since around 2003, Chinese patients a decade later still paid a large share of the costs of health care out of pocket.^16^, ^17^, ^20^, ^21^ Thus financial barriers to health care remained significant and both socio‐demographically and socio‐economically patterned.^19^, ^22^, ^23^, ^24^ Co‐payment rates and reimbursement ceilings privileged those already privileged: urban employees, for example, enjoyed lower co‐payment rates and higher reimbursement than urban residents outside the labour force and rural dwellers.^17^ Restrictions on the portability of benefits made it hard for internal migrants, who constitute about 10% of the population, to use their insurance.

In addition, incentives to generate revenues contributed to ‘unethical’ practices and problematic relationships between doctors and patients.^18^, ^25^, ^26^, ^27^ These incentive structures emerged as a side‐effect of the retrenchment of government financing for health care accompanied by continuing government control over the prices of some services and the wages of medical staff.^16^, ^18^ Unethical practices, which seem mainly to be aimed at extracting additional payments from patients, include prescribing more expensive medicines than strictly necessary, requiring unnecessary diagnostic tests and accepting informal payments (*hong bao*), often in advance of surgical procedures.^26^

Despite these problems, little is known about the Chinese public's satisfaction with the health‐care system. Anecdotally, public confidence in the system is said to be very low.^28^, ^29^ However, there have been few published nationwide surveys focussing on satisfaction and related attitudes. Extant surveys focus mainly on monitoring public health and insurance provision. The largest national survey on health (the National Health Services Survey) has been conducted by the Ministry of Health every 5 years since 1993, but the questions on satisfaction are addressed to patients and focus on specific experiences rather than the health‐care system as a whole. Only one other study on public satisfaction with the health‐care system is known to have been conducted, by the Ministry of Health and Peking University in 2010–2011, but like many Chinese government‐sponsored surveys, it is not publicly available for analysis and only limited information on the results has been published.^30^ Chinese health policy as it evolved up until the 2000s appears to have largely ignored popular preferences, and there is limited freedom of discussion in the media.^31^ The most recent round of health‐care reforms in 2009 featured a public consultation process, but there was no patient lobby and officials and experts still dominated policymaking.

## Study design

Our study is based on detailed and systematic analysis of a single nationwide survey. The Research Center for Contemporary China at Peking University carried out fieldwork on our behalf between 1 November 2012 and 17 January 2013. Our survey was designed to represent the target population of Chinese citizens aged 18–70 residing for more than 30 days in family dwellings in all provinces of mainland China. For a summary of the procedures for constructing the survey instrument and sampling methodology, see Appendix S1.

Our survey asked respondents: ‘In general, would you say you are very satisfied, fairly satisfied, fairly dissatisfied or very dissatisfied with the way health care is run in our country?’ This is similar in form to the World Health Survey question used by Bleich *et al*.^7^: ‘In general, would you say you are very satisfied, fairly satisfied, neither satisfied nor dissatisfied, fairly dissatisfied or very dissatisfied with the way health care runs in your country’. The only difference is that our question used a four point scale instead of a five point scale, as we wished to discourage neutral answers. Both questions are designed to elicit a response to the overall state of the health‐care system in the country.

Unlike the surveys on which most previous studies of health‐care system satisfaction have been based – Bleich *et al*.^7^ is the exception – our survey was explicitly designed to probe attitudes towards the health‐care system. We are thus able to include independent variables that enable us to test the full range of explanations identified in previous studies. To test the effects of socio‐demographic variables, we examine (rural vs. urban) residence, age and education as well as gender. In addition, because in China people's household residence registration (*hukou*) determines the types of insurance schemes available to them, we include indicators for whether the respondent has an agricultural or non‐agricultural registration, and whether their household registration is local or non‐local. Second, we include questions on self‐rated physical and ‘emotional’ (mental) health. Third, to test the effects of financial barriers to health care, we examine health insurance status (whether or not people have some form of insurance), whether people think their insurance is adequate, and income. Our instrument asked whether respondents had any one of nine common types of insurance including the three main social insurance types and employer‐provided as well as individually purchased private insurance. We define as ‘insured’ any respondent having at least one type of insurance. Fourth, to test the associations between health‐care system satisfaction and ideological beliefs, we include questions on whether individuals should pay for their own health‐care costs and perceptions of inequality in access to care. Given the lively debates in China over fairness and equality in health‐care provision, we think it is appropriate to examine ‘egalitarian’ ideological beliefs as Missinne *et al*.^9^ did in Europe. Fifth, to test utilization effects, we include the numbers of visits to hospitals and, separately, to clinics, in the last year. Sixth, to test the effects of media use, we use two measures: frequency of using social media including the internet, mobile phones and networking sites to get news, and frequency of watching television news.

Finally, we include scales to test how perceptions of services are associated with health‐care system satisfaction. Because evaluations of services in primary care institutions and hospitals loaded on different factors in exploratory factor analysis, we include separate scales for perceived competence, convenience and value for money in both types of institutions. (Health‐care surveys sometimes use physical distance from the nearest health‐care facility as a proxy for convenience. However, this neglects the fact that difficulties presented by distance can vary according to respondents’ state of health, available modes of transport, traffic congestion and other conditions, so we chose to use a direct subjective evaluation of convenience.) As there are strong correlations among evaluations of different kinds of primary care institutions, we use the term ‘clinic’ as a generic term for several different types, including township, town or street health service centres, community health service stations, village clinics (*cun weisheng shi*) and small clinics (*zhensuo*). Because unethical practices are perceived to be prevalent in Chinese hospitals^26^ and have been noted as a phenomenon in other contexts, too,^32^, ^33^ we asked about three types – unnecessary diagnostic testing, prescribing more expensive medicines than needed and taking informal payments. As the three measures correlate, we include a scale for their perceived likelihood. The complete list of independent variables, along with descriptive statistics, the percentage of ‘don't know’ and ‘no answer’ responses, and the range of each variable are summarized below in Table 1. Details on question wording, exploratory factor analysis and scale construction are given in Appendix S2.

**Table 1:** Independent variables and their multivariate associations with health‐care system satisfaction

	Valid, %	Descriptive statistics				Odds ratio	Multivariate analysis		
		Mean	Std. dev.	Valid, *N*	Dk/Na, %		95% CI		*P*‐value
							Lower	Upper	
Socio‐demographics									
Male	50	Na	Na	3680	0	1.00	0.85	1.17	0.976
Age				3680	0				
Middle (30–59)	61	Na	Na			1.00			
Young (18–29)	28	Na	Na			1.05	0.74	1.48	0.799
Old (60+)	11	Na	Na			1.40	1.07	1.84	0.014
Education				3680	0				
Primary only	30	Na	Na			1.00			
Junior high education	34	Na	Na			1.08	0.82	1.42	0.596
Senior high/technical	26	Na	Na			0.82	0.60	1.12	0.222
University	10	Na	Na			1.07	0.70	1.62	0.771
Rural residence	45	Na	Na	3680	0	1.25	0.87	1.81	0.227
Non‐agricultural residence registration	37	Na	Na	3680	0	0.86	0.67	1.11	0.257
Local residence registration	89	Na	Na	3680	0	1.36	0.93	1.99	0.116
Self‐reported health									
Self‐assessed physical health				3670	0.3				
Poor or very poor physical health	11	Na	Na			1.00			
Average health	18	Na	Na			1.30	0.86	1.98	0.214
Good health	49	Na	Na			1.49	1.02	2.17	0.039
Very good health	22	Na	Na			1.75	1.10	2.77	0.018
Emotional health				3664	0.4				
Poor or very poor emotional health	5	Na	Na			1.00			
Average emotional health	19	Na	Na			1.18	0.68	2.03	0.559
Good emotional health	51	Na	Na			1.36	0.79	2.36	0.271
Very good emotional health	25	Na	Na			1.21	0.68	2.18	0.518
Financial access									
Has health insurance	92	Na	Na	3680	0	**1.76**	**1.21**	**2.55**	**0.003**
Adequacy of insurance coverage^a^				3142	15				
Does not suit my needs	23	Na	Na			1.00			
Suits my needs quite well	70	Na	Na			**4.45**	**3.48**	**5.70**	**0.000**
Suits my needs very well	7	Na	Na			**8.16**	**4.63**	**14.37**	**0.000**
Income^a^				2772	25				
Lowest	25	Na	Na			1.00			
Second lowest	24	Na	Na			1.32	0.92	1.89	0.140
Middle	23	Na	Na			1.39	0.98	1.97	0.064
Second highest	18	Na	Na			1.18	0.83	1.68	0.348
Highest	10	Na	Na			1.21	0.81	1.82	0.357
Ideological beliefs									
We should pay for own health care^a^				3288	11				
Disagree	40	Na	Na			1.00			
Somewhat agree	51	Na	Na			**1.52**	**1.20**	**1.92**	**0.000**
Strongly agree	9	Na	Na			**2.19**	**1.39**	**3.47**	**0.001**
Extent of inequality in access^a^(1 least…4 most)	*Na*	*2.93*	*0.61*	*3481*	*5.0*	**0.54**	**0.40**	**0.72**	**0.000**
Extent of inequality in access * rural context	Na					1.29	0.92	1.80	0.138
Utilization									
N hospital visits over last year	Na	0.49	0.94	3680	0	**0.75**	**0.65**	**0.87**	**0.000**
N hospital visits * Rural context	Na					1.30	1.05	1.61	0.015
N clinic visits over last year	Na	1.51	2.04	3680	0	1.03	0.97	1.10	0.323
Media use									
Uses social media for news^a^ (1 never…5 daily)	*Na*	*2.12*	*1.26*	*3638*	*1.0*	**0.78**	**0.69**	**0.89**	**0.000**
Watches TV for news^a^ (1 never…5 daily)	Na	4.11	1.20	3680	0.9	1.06	0.96	1.18	0.259
Perceptions of services									
Convenience of hospitals^a^ (1 worst…4 best)	*Na*	*2.46*	*0.76*	*3083*	*16*	1.22	1.02	1.46	0.029
Convenience of clinics^a^ (1 worst…4 best)	*Na*	*3.43*	*0.57*	*3282*	*11*	1.05	0.85	1.29	0.651
Clinics: value for money^a^ (1 worst…4 best)	*Na*	*2.84*	*0.58*	*3039*	*17*	1.26	1.03	1.53	0.024
Hospitals: value for money^a^ (1 worst…4 best)	*Na*	*3.36*	*0.49*	*2870*	*22*	1.24	1.04	1.49	0.016
Clinics: competence^a^ (1 worst…4 best)	*Na*	*2.74*	*0.55*	*3253*	*12*	1.11	0.85	1.47	0.444
Hospitals: competence^a^ (1 worst…4 best)	*Na*	*3.36*	*0.49*	*3084*	*16*	1.22	0.93	1.60	0.156
Likelihood of unethical practices^a^ (1 least…4 most)	*Na*	*2.59*	*0.61*	*3427*	*7.0*	**0.63**	**0.52**	**0.76**	**0.000**

Model fit statistics ‐2LL fitted: 193 326; null: 379 804; difference: 186 479, d.f. = 31 (*P* < 0.001).

Bold: *P* < 0.01; Na: not applicable; Italics: covariates; Roman type: factors.

a Further details on scale construction are in Appendix S2.

Two limitations of the study design should be acknowledged at the outset. As it relies on a cross‐sectional survey, it cannot demonstrate that the associations we have found involve causal relationships. For socio‐demographic and health measures, as well as some variables related to access, utilization and media use, it seems safe to assume that health‐care system satisfaction is the effect and not the cause, but for ideological beliefs and perceptions of services, it may be just as plausible to assume that causality runs the other way, or both ways. We have used causal language because our methodology requires the assumption that some variable is dependent, but we acknowledge that association is not causation. Second, we do not know enough about Chinese respondents’ expectations to understand exactly what they mean when they say they are ‘satisfied’, and to what extent their satisfaction levels are comparable on a cross‐national basis.

## Method of analysis

To determine which factors are most closely associated with health‐care system satisfaction, we ran a series of multilevel logistic regressions on a dichotomized version of the dependent variable in which a value of zero represents dissatisfaction and a value of one represents satisfaction.^8^ We used a multilevel form of logistic regression because respondents are clustered by primary sampling units (PSUs) constituting a random sample of county‐level administrative units across China. Only one variable, whether it is an urban or rural location, is measured at the level of the PSU, and all other variables are measured at individual level. We ran the regression once for each individual variable on a bivariate basis (Appendix S3). Then, to test whether the bivariate effects are conditioned by other variables, we ran the regression once for all the variables together (Table 1). To test whether attitudinal measures were masking other effects of substantive interest, we also reran the analysis using a conventional set of socio‐demographic and employment indicators with no attitudinal measures except self‐assessed health (Appendix S4). To control for collinearity, we computed tolerance statistics for all the independent variables in the model. All had variance inflation factors less than 2.0, well within the 2.5 limit which Allison suggests as a guideline indicating possible cause for concern.^34^

The median level of missing data for observed indicators was 15%. In multivariate analysis, casewise omission of missing data would result in huge data loss, and if the data are not missing completely at random, it would also result in biased estimates. We therefore used multiple imputation methods to create five complete data sets and pooled the results to obtain parameter estimates.^35^, ^36^ For income, where missing data were as high as 25%, we also reran the analysis using only respondents who gave their income to check that the significance of the parameters had not been affected by imputation.

Because expectations of health‐care systems may differ between urban and rural areas, we tested systematically for the interaction of rural context with the other variables. We considered the possibility of using county‐level measures of health expenditure and service provision as independent variables but we found that high quality county‐level data on these items were not publicly available. County‐level data are available for numbers of health‐care professionals and numbers of beds per head of population, but neither proved significant in exploratory analyses.

The parameter estimates, computed using Mplus software, take account of the clustered structure of the data by administrative unit, and apply case weights. To reduce the possibility of interpreting spurious results, we focus on parameters which are significant at 0.01 level or less, and treat those significant at more than 0.01 but less than the 0.05 level as marginal.

## Results

**Figure 1:**
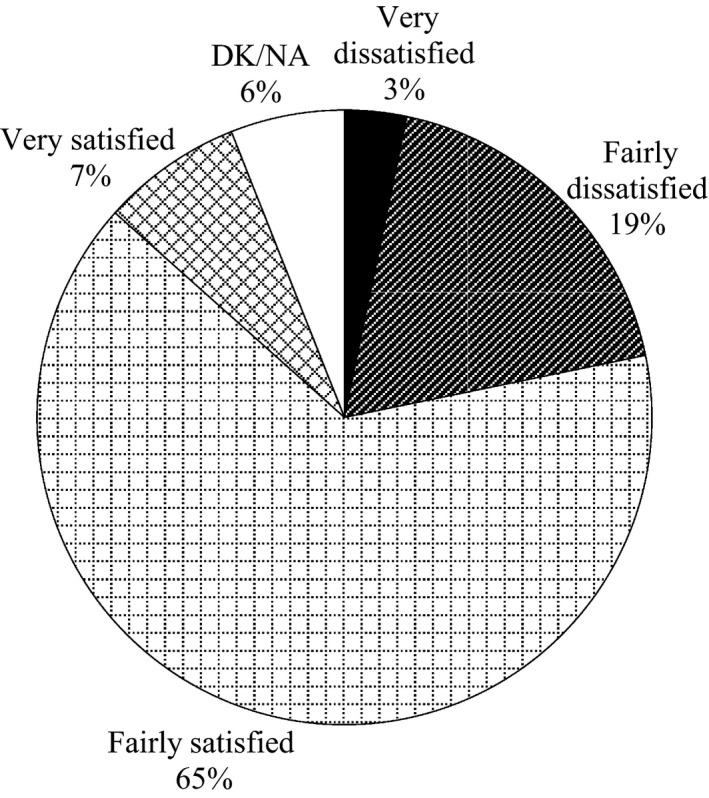
Satisfaction with the Chinese health‐care system. C1. In general, would you say you are very satisfied, fairly satisfied, fairly dissatisfied or very dissatisfied with the way health care is run in our country? Source: China National Health Attitudes Survey, 2012–2013, fieldwork 1 November 2012–17 January 2013, *N* = 3680.

Responses to our question on satisfaction showed 7% were ‘very satisfied’ with the way the health‐care system is run, 65% ‘fairly satisfied’, 19% ‘fairly dissatisfied’, 3% ‘very dissatisfied’ and 6% said ‘don't know’ or gave no answer (Fig. 1). The overall satisfaction level of 72% (=65 + 7) is slightly higher than the 69% reported in the Ministry of Health/Peking University survey conducted between 10 December 2010 and 1 February 2011,^30^ but as sampling error was around 3% for both surveys, satisfaction rates in the two surveys are very similar.

Bivariate analyses (Appendix S3) identify positive associations (all *P* < 0.01) between satisfaction and being age 60 or older, rural residence, good physical health, good or very good emotional health, having health insurance, having insurance which meets the respondent's needs well or very well, agreement or strong agreement that people should take personal responsibility for health‐care costs, as well as perceptions of convenience, value for money and competence in hospitals and clinics. Bivariate analyses also identify negative associations (all *P* < 0.01) between satisfaction and having a junior high school education, only average emotional health, the perceived extent of inequality in access, the number of hospital visits over the last year, use of social media for news and the perceived likelihood of unethical practices. Gender and income have no significant bivariate associations with health‐care system satisfaction.

In our multivariate analysis (Table 1), among the socio‐demographic variables, only older age and good self‐reported physical health remain significant, and the associations are weaker. Gender, education and rural residence are not significant. Likewise, type and location of residence registration are not significant. As it has been argued that rural Chinese are more tolerant of inequality, because they have never had the benefit of comprehensive state‐backed welfare guarantees,^37^ and therefore are more likely to endorse the current health‐care system in spite of inequalities, we report the parameters for this interaction effect even though it is not significant.

In terms of understanding the influence of financial barriers to care, we find that having health insurance is associated with a 76% increase in the odds of being satisfied (*P* < 0.01). If insurance is perceived to suit the respondent's needs ‘well’ or ‘very well’ the odds increase by more than four times. We detect no income‐related effects, and we find the same if we rerun the analysis excluding all cases where income data were imputed.

In terms of ideological beliefs, we find that agreeing with the idea that people have a personal responsibility for meeting health‐care costs is associated with a 52% increase in the odds of satisfaction if agreement is moderate (*P* < 0.001), and the odds more than double for strong agreement. In addition, each one‐unit increase in the perceived extent of inequality in access reduces the odds of satisfaction by 46% (*P* < 0.001).

In terms of utilization, each hospital visit over the last year reduces the odds of satisfaction by 25% (*P* < 0.001), whereas clinic visits have no significant effect. The interaction effect of hospital visits with rural residence, which is at the margin of significance (*P* < 0.05), suggests that utilization is negatively associated with health‐care system satisfaction mainly in urban areas.

As far as media effects are concerned, watching television, the main source of news for the vast majority of Chinese people, has no significant effect. However, relying on social media for news has strong negative associations with satisfaction, reducing its odds by 22% (*P* < 0.001).

In terms of perceptions of services, the perceived likelihood of unethical practices has a strong association with health‐care system satisfaction, reducing its odds by 37% (*P* < 0.001). Perceptions of value for money for both clinics and hospitals and convenience of hospitals have positive associations at the margin of significance.

## Discussion

Our analysis suggests that socio‐demographic characteristics (typically seen as affecting people's expectations) are much less important than some previous studies have indicated.^7^, ^8^, ^9^, ^10^ However, this is most likely because our analysis includes a rich array of attitudinal variables whereas most previous studies do not. The results from our supplementary analysis (Appendix S3), where all attitudinal variables except self‐assessed health are excluded, are much more consistent with the prior research. Thus, old age and health are strong positive influences (*P* < 0.001), consistent with European results.^7^, ^9^, ^11^ Rural residence is a marginal positive influence (*P* < 0.05), consistent with results from the former Soviet Union.^8^ Consistent with Bleich *et al*.,^7^ those with most and least education are more satisfied than those with a high school education (*P* < 0.05). Consistent with most prior studies,^7^, ^8^, ^10^, ^11^ gender is not significant.

Some previous studies indicate that financial barriers to access are associated with reduced satisfaction.^10^, ^11^, ^12^, ^13^ Our results support this. Although we find income to have no effect, our data show that health insurance – which reduces financial barriers – has a positive relationship with health‐care system satisfaction, as does reported adequacy of insurance. As social insurance schemes are a principal policy lever, we also ran a check on whether the type of insurance that people have matters, controlling for occupation (see Appendix S4). Even though the three principal types of health insurance provide different levels of benefits, all types increase satisfaction (*P* < 0.05), and these effects are robust even when perceived adequacy of insurance is introduced as an additional control. The fact that the perceived adequacy of insurance is highly significant in Table 1 underlines that there is a subjective element to financial access, and the perceived need for insurance varies across households.

Given China's limited health insurance provision, it is surprising that we find no association between income and satisfaction so that (on this issue) China resembles Europe^9^ rather than the United States.^13^ Income appears to matter neither in the multivariate model (Table 1), nor in bivariate models (Appendix S3), nor in the supplementary analysis where perceived adequacy of insurance is not controlled (Appendix S4). When we regress the same independent variables used in the supplementary analysis on a four point scale measuring how easily the household is able to afford its medical bills, the income quintile dummies are all in the expected direction and highly significant (*P* < 0.001), so we do not think that the income measure is invalid. One possibility is that most respondents do not actually think about the financial implications of the way the health‐care system is run until they or a family member get sick. However, when we rerun the supplementary analysis selecting only those 48% of respondents living in households where a household or close family member used hospitals in the last year, we do not find that income matters any more than when all respondents are included. Similarly, if we restrict the analysis to the 28% of respondents who have themselves visited hospitals for their own health in the past year, income is still insignificant. Perhaps respondents adjust their expectations according to their income level, which means that people on different income levels who give the same health‐care system satisfaction rating are evaluating the system using different standards. Prior research suggests income effects are complex and difficult to explain in any context, and our results support this.

Our analysis calls to mind research showing that household registration status (*hukou*) conditions access by affecting eligibility for health insurance.^19^, ^22^, ^23^ Although having local registration and non‐agricultural registration are not significant in the multivariate model (Table 1), the supplementary analysis shows that local registration increases the odds of satisfaction by 61% (*P* < 0.05, Appendix S4) when attitudes are not controlled. If we introduce a control for adequacy of insurance, local registration still matters with the same sign (*P* < 0.05), and if we introduce the significant attitudinal variables in Table 1 one at a time local registration only gradually loses significance, suggesting that it matters not for any one reason, such as by affecting people's ability to use their insurance, but for a combination of reasons.

Our finding that ideological beliefs are associated with satisfaction – and that people who accept personal responsibility for their own health care are more satisfied – resonates with research from Europe. Missinne *et al*.^9^ found Europeans with egalitarian beliefs to be less satisfied, and thus non‐egalitarians to be more satisfied. Further research is needed, however, to understand the interaction between ideology and other factors. Actual and perceived health‐care system trends – towards more equitable provision or towards more unegalitarian personal responsibility – might for example shape the relationship between ideology and satisfaction.

The fact that perceptions of the health‐care system as unequal are associated with lower satisfaction underscores the importance of looking at perceptions of reality as well as normative values when we seek to understand how ideological beliefs affect satisfaction with health‐care systems. We have gone beyond Missinne *et al*.'s study^9^ in measuring the effects of perceptions as well as values.

The negative coefficient for utilization in China echoes similar findings in the former Soviet Union.^8^ Given that health‐care systems designed for centrally planned economies were placed under severe strain across the post‐Soviet space and transitional governments were generally unprepared to deal with these challenges, China's negative utilization effect does not speak well of China's health‐care achievements. It may reflect diverse problems which are not otherwise captured in the model, including overcrowding due to the fact that most patients self‐refer to city hospitals.^17^, ^19^

Our study confirms the speculation of previous studies that the tone of media coverage can affect health‐care system satisfaction.^7^, ^8^ The fact that using the much less well‐controlled social media for news reduces satisfaction, implies either that these media are spreading negative rumours about the health‐care system, or that they are telling the truth about problems that mainstream media neglect. The fact that the Chinese government makes vigorous efforts to regulate online behaviour and content,^38^ and rewards social network providers for taking a pro‐government stance on sensitive issues^39^ does not seem to have prevented the spread of negative attitudes towards the health‐care system among social media users.

Our study confirms that perceptions of services matter, in line with the results of prior studies.^7^, ^11^, ^12^ We have improved on those studies by offering systematic tests of a range of service dimensions across both primary care and hospitals. The fact that convenience of hospitals and perceived value for money of clinics and hospitals are marginal positive influences (*P* < 0.05) is consistent with a common sense notion of what health‐care systems are supposed to deliver. In addition, our study is the first to measure the impact of another service dimension – the perceived likelihood of unethical practices. The fact that this has the strongest effect among all the measures of perceptions of services we considered (*P* < 0.001) underlines how sensitive an issue unethical practices have become in the Chinese context.

This study has a number of limitations. First, because there are little published data on health service provision at the level of our PSUs, we have not been able to comment on how local provision correlates with satisfaction, even though we know there can be substantial variations in the ways different Chinese cities and counties implement central government health policy. Second, we do not yet have sufficient comparable trend data to comment on temporal aspects of satisfaction levels, or how satisfaction has changed in response to particular policy initiatives, whether at central or local level. Our study does, however, provide robust and important findings on which future research can build.

## Conclusions

In the introduction to this article, we argued that public satisfaction research is undertheorized. Having systematically tested a range of competing explanations, we can now demonstrate that at least in China, having health insurance and the adequacy of that insurance are key to satisfaction. Furthermore, in a context where there is little consensus about who should pay for care, ideology has important effects, with citizens being cross‐pressured by the perceived level of inequality in access and belief in personal or household responsibility for meeting one's own costs. How citizens learn about the health‐care system matters, too, as demonstrated by the effects of utilization and media use on satisfaction. Finally, the perceived likelihood of unethical medical practices severely reduces satisfaction, at least in countries like China where such problems are salient. Further research is needed to test these conclusions in other contexts so that we can delineate more precisely under what circumstances they apply.

For China specifically, the findings have important policy implications. First, policies to extend insurance, such as those that led to the achievement of more than 90% coverage by the time of our survey, appear to increase public satisfaction. More needs to be done, however, to make sure that insurance meets people's needs. More extensive coverage and easier transferability of benefits may improve perceptions about equality of access and so increase satisfaction.

Second, governments must do more to rebuild the public's fiduciary relationship with the medical profession. Recent reforms have tried to restructure incentives and rebalance hospitals and medical professionals’ priorities so that patients’ interests come first, but our data show that at the time of survey suspicions of unethical practices damaged health system satisfaction more than any other single dimension of health‐care system performance.

Third, the negative influence of social media use on health‐care system satisfaction is symptomatic of a policy process that has, historically, failed to take adequate account of popular preferences or to allow open and inclusive public discussion. Control over the mass media has not prevented adverse publicity but has simply moved negative stories online. While we acknowledge progress on this over the past decade, particularly since the SARS epidemic, we think it is important that Chinese health‐care policymakers continue to open up the process and allow more public participation in designing and implementing health policy.

## Conflict of interest

None.

## Source of funding

This study benefited from the financial support of the project ‘Performance Evaluations, Trust and Utilization of Health Care in China’, grant number ES/J011487/1, by the UK Economic and Social Research Council, Swindon, UK.

## Supporting information


**Appendix S1.** Instrument design and survey methodology.
**Appendix S2.** Questions, coding and scale construction procedures.
**Appendix S3.** Bivariate associations with health‐care system satisfaction.
**Appendix S4.** Supplementary analysis: social structure, employment and insurance type.
