# Supplementary material for: Explaining public satisfaction with health‐care systems: findings from a nationwide survey in China
Source: Health Expect. 2015 Nov 23;19(3):654–66. doi: 10.1111/hex.12429 (PMC4991297; doi:10.1111/hex.12429)
Supplement: Supplementary file 1 — Appendix S1. Instrument design and survey methodology. Appendix S2. Questions, coding and scale construction procedures. Appendix S3. Bivariate associations with health‐care system satisfaction. Appendix S4. Supplementary analysis: social structure, employment and insurance type. [file HEX-19-654-s001.docx]

**Appendix S1. Instrument Design and Survey Methodology**

At the start of our research, we commissioned nine focus groups with between seven and nine participants each from a mix of urban and rural backgrounds in three cities at different levels of development in northern, southern and central China. We drafted the discussion guide in English with our co-Investigators, and native speakers from RCCC translated it into Chinese and moderated the discussions without any non-Chinese present. We analysed the transcripts to better understand what kind of language respondents used to talk about health care institutions. We then drafted our questionnaire, again in English, drawing where possible on the wording of questions from existing China health surveys. We asked native Chinese speakers with expertise in the Chinese health system to translate it into modern standard Chinese, back-translated it into English, and discussed question wording at length with our translators. We found that concepts of performance evaluation (“satisfaction”) were easily conveyed in everyday language and did not cause translation difficulties. RCCC then piloted the survey near Beijing with a sample of 50 respondents, around half of whom had “non-agricultural” residence registration and around half of whom had “agricultural” residence registration. On the basis of the responses as well as feedback from the interviewers, we revised the instrument again, simplifying some questions to minimize respondent fatigue.

The nationwide sample was constructed as follows. At the first stage, the country was divided into three official macro-regions: Eastern, Central and Western, and each macro-region into urban and rural areas. At the second stage, 60 primary sampling units (PSUs) corresponding to counties, county-level cities or urban districts were randomly selected with probability proportionate to population. At the third stage, three half-square minutes (HSMs) of latitude and longitude were chosen with probabilities proportionate to population density, and within each of these, again proportionate to population density, several spatial square seconds (SSS) corresponding to 90m x 90m squares were selected at random. Within each SSS, all dwellings were enumerated, and 27 were chosen in each HSM by systematic sampling. Interviewers, who were local college students supervised by RCCC fieldworkers, visited each selected dwelling. Within each they enumerated the inhabitants and identified one qualified respondent using the Kish method, making several call-backs if the respondent was not at home. The sample covered 5,424 dwellings, in which 3,680 valid interviews were conducted giving a response rate of 67.8%, and a margin of error of plus or minus 3%. The survey team applied post-stratification weighting to match the 2010 census distributions by gender and age classified into six deciles, 18-29, 31-39, 41-49, 51-59 and over 60 years old, as detailed below.

|  | Census 2010 | | Unweighted | | Weighted | |
| --- | --- | --- | --- | --- | --- | --- |
|  | Female | Male | Female | Male | Female | Male |
| Age | % | % | % | % | % | % |
| 18-29 | 27∙7 | 27∙3 | 17∙3 | 18∙6 | 27.7 | 27.3 |
| 30s | 21.8 | 22.0 | 19.6 | 18.4 | 21.7 | 22.0 |
| 40s | 23.4 | 23.5 | 24.5 | 24.0 | 23.4 | 23.5 |
| 50s | 16.3 | 16.3 | 19.0 | 18.4 | 16.3 | 16.3 |
| 60+ | 10.9 | 10.9 | 19.5 | 20.7 | 10.9 | 10.9 |
| Sources: China National Health Attitudes Survey, nationwide survey in mainland China, fieldwork 1 November 2012-17 January 2013, N=3,680. Census Office of the State Council, National Bureau of Statistics Population and Employment Statistics Division*. Census of the People’s Republic of China* (Vol. 1). Beijing：China Statistics Press; 2012, Table 3-1a. | | | | | | |

Further details on the process of instrument design and survey methodology as well as a bilingual version of the questionnaire can be found on our project web site: http://www.glasgow.ac.uk/petu

**Appendix S2. Questions, coding and scale construction procedures**

Health care system satisfaction: *C1. In general, would you say you are very satisfied, fairly satisfied, fairly dissatisfied or very dissatisfied with the way health care is run in our country?* (4-point scale recoded to run from 1 very dissatisfied to 4 very satisfied).

SOCIO-DEMOGRAPHICS

Gender: interviewer coded.

Age: computed from *A1. In which year were you born?*

Education: coded from *A3a. What is your highest level of education?* (9-point scale collapsed into four categories: primary or less, junior high education, senior high/technical, and university).

Rural residence: coded according to administrative categorization of PSU as a county (*xian*).

Non-agricultural residence registration: coded from *A4. Do you have an agricultural or non-agricultural hukou?*

Local residence registration: coded from *A5. Where is your current hukou?* (five possible answers: this village/urban district, another village/urban district in this town/city, another town/urban district in this county/city, another county/city in this province, another province/city). All residents with hukou in the same city or county where they live are classified as local.

SELF-REPORTED HEALTH

Self-assessed physical health: *B1. Over the past 12 months, what do you think your physical health is?* (5-point scale collapsed into four categories: poor or very poor physical health, average health, good health, very good health)

Emotional health: *B2. Over the past 12 months, what do you think your emotion health is?* (5- point scale collapsed into four categories: poor or very poor emotional health, average emotional health, good emotional health, very good emotional health).

FINANCIAL ACCESS

Has health insurance: *B16. Are you currently the policy holder/primary beneficiary of any of the types of health insurance listed below?* (Nine types listed; respondents having any one of the nine types are classified as insured).

Adequacy of insurance coverage^:^ *B19. Considering your life circumstances and overall state of health, how well do you think your main insurance policy meets your needs?* (4-point scale collapsed into three categories: does not suit my needs, suits my needs quite well, suits my needs very well).

Income: Coded from two questions: *D13. Please estimate your family's total income last year from all sources (including all household members' salaries, bonuses, subsidies, dividends, interest, insurance payments, pensions, commercial profit, rent, interest, gifts etc.);* and (if respondent does not answer D13) *D13a. Please estimate your family's total income last year from all sources, whether is it more or less than 60,000?* (Then prompt respondents to choose a position on a 12-point scale from 10,000 or less to 300,000 or more; respondents at the bottom of the scale are coded 10,000; respondents at the top of the scale are coded 300,000; and all others are coded to the midpoint of their scale position). Answers to D13 and D13a are then combined to produce an estimate of total family income. This estimate is then equivalized by dividing it by the number of household members, counting the first adult as one, other adults as 0.5 and children and adults over seventy as 0.3. Missing data (25% of cases) are multiply imputed and then cases are allocated to income quintiles.

IDEOLOGICAL BELIEFS

We should pay for own health care: *C6a. Please tell me whether you strongly agree, somewhat agree, disagree, strongly disagree with the following statements: We should pay by ourselves for the cost of our own health care.* (4-point scale, collapsed into three categories: disagree, somewhat agree, strongly agree)

Extent of inequality in access: coded from *C3_1. Firstly, we would like to ask about the extent of equality in access to health care. Compare the health care situation between different groups of people, would you say there is a lot of inequality, some inequality, fair amount of equality or a great deal of equality in access to health care between: rich and poor people, urban and rural residents, civil servants and ordinary people, men and women, migrants from other provinces and local people?* (4-point scale recoded to run from 1 very equal to 4 very unequal). In confirmatory factor analysis, perceived inequality between rich and poor, civil servants and ordinary people and urban and rural residents form a single factor with loadings of 0.84, 0.84 and 0.81, and so we average these three items to form a scale (Cronbach’s alpha=0.81).

UTILIZATION

N hospital visits over last year: coded from *B10_1. Over the past 12 months, have you used any of the following types of health services for your own health problems? (Show cards, ask one by one)* and *B10_2. (For each type of health service used) how many times did you use the service in the last 12 months?* We count all visits to three levels of hospitals: county/city/district hospitals, prefecture-level hospitals and provincial hospitals.

N clinic visits over last year: coded from B10_1 and B10_2 as above. We count all visits to four types of clinics: small clinics (*zhensuo*), village health clinics, community health service stations, and township, town or street health centres or health services centres.

MEDIA USE

Uses social media for news: coded from *C4. About how often do you: Read news through mobile phone, Use the internet to find out news, Use social networking sites to find out news* (5-point scale recoded to run from 1 never to 5 every day). In confirmatory factor analysis, frequency of use of internet, mobile telephones and social networking sites form a single factor with loadings of .88, .86 and .85 respectively, and so we average these three items to form a scale (Cronbach’s alpha=.85).

*Watches TV for news:* coded from C4 as above (5-point scale from 1 never to 5 every day).

PERCEPTIONS OF SERVICES

Convenience of hospitals: Coded *from C24. How would you rate the following types of hospitals or clinics by the criteria specified below: skills and experience of doctors, value for money and convenience?*  (4-point scale recoded to run from 1 very bad to 4 very good). Respondents were asked to rate each of eight types of facilities by all three criteria. The types of facilities were: small clinics, village health clinics, community health service stations, township, town or street health centre or health services centres, county/city/district hospitals, city/prefecture hospitals and province-level hospitals. In a confirmatory factor analysis of ratings for all eight types of facilities by all three criteria, convenience ratings for the three levels of hospitals form a single factor with loadings of 0.89, 0.82 and 0.77 respectively. We therefore construct a scale by averaging these three convenience ratings (Cronbach’s alpha=0.83).

Convenience of clinics: coded from C24 as above. In the confirmatory factor analysis, convenience ratings of small and village clinics, community health service stations, and town or township health service centres form a single factor with loadings of 0.84, 0.82, 0.80 and 0.68 respectively. We thus construct a scale by averaging these four convenience ratings (Cronbach’s alpha=0.85).

Clinics value for money: coded from C24 as above. In the confirmatory factor analysis, value for money ratings of small and village clinics, community health service stations, and town or township health service centres form a single factor with loadings of 0.85, 0.84, 0.82 and 0.72 respectively. We construct a scale by averaging these four value for money ratings (Cronbach’s alpha=.91)

Hospitals value for money: coded from C24 as above. In the confirmatory factor analysis, ratings of value for money in county/city/district hospitals, city/prefecture hospitals and province-level hospitals form a single factor with loadings of 0.92, 0.91 and 0.81 respectively. We average these value for money ratings to form a scale (alpha= .93).

Clinics competence: coded from C24 as above. In the confirmatory factory analysis, ratings of skills and competence of doctors at small and village clinics, community health service stations, and town or township health service centres form a single factor with loadings of 0.87, 0.83, 0.82 and 0.71 respectively. We average these four skills and competence ratings to form a scale (Cronbach’s alpha=0.85).

Hospitals competence: coded from C24 as above. In the confirmatory factor analysis, ratings of skills and experience for doctors in county/city/district hospitals, city/prefecture hospitals and province-level hospitals form a single factor with loadings of 0.89, 0.85 and 0.73 respectively. We average these three skills and competence ratings to form a scale (Cronbach’s alpha=0.85).

Likelihood of unethical practices: coded from *C21.* *Some people say medical ethics is a big problem in this country, but others say such reports are just exaggerated. Judging from your own personal experience, how likely do you think it is that you would encounter the following types of situations in city or county hospitals around here? Prescribing medicines not covered by insurance even when effective alternatives covered by insurance are available; Taking bribes (“red envelopes” or hong bao) for treatment which has already formally been paid for; Requiring comprehensive check-ups from patients even when the diagnosis is perfectly clear; Selling fake medicines.* (4 point scale from 1 very unlikely to 4 very likely). In a confirmatory factor analysis, the first three items load on a single factor with loadings of 0.78, 0.78 and 0.74 respectively, and so we average them to form a scale (Cronbach’s alpha= 0.73).

Further details of the survey including the full questionnaire can be found at http:// www.glasgow.ac.uk/petu

| **Appendix S3. Bivariate associations with health care system satisfaction** | | | | | |
| --- | --- | --- | --- | --- | --- |
|  | Odds | 95% CI | | | p- |
|  | Ratio | lower | - | upper | value |
| SOCIO-DEMOGRAPHICS | | | | |  |
| Male | 1.03 | 0.90 | - | 1.18 | 0.696 |
| Age |  |  |  |  |  |
| Age 30-59 | 1.00 |  |  |  |  |
| Young (Age-18-29) | 0.84 | 0.65 | - | 1.08 | 0.176 |
| Old (Age 60+) | **1.35** | **1.09** | **-** | **1.68** | **0.007** |
| Education |  |  |  |  |  |
| Primary only | 1.00 |  |  |  |  |
| Junior high education | 1.25 | 1.00 | - | 1.56 | 0.051 |
| Senior high/technical | **0.72** | **0.59** | **-** | **0.88** | **0.002** |
| University | 0.91 | 0.68 | - | 1.23 | 0.550 |
| Rural location | **1.70** | **1.15** | **-** | **2.52** | **0.008** |
| Non-agricultural residence registration | 0.79 | 0.63 | - | 0.97 | 0.027 |
| Local residence registration | **1.70** | **1.21** | **-** | **2.39** | **0.002** |
| SELF-REPORTED HEALTH |  |  |  |  |  |
| Self-assessed physical health |  |  |  |  |  |
| Poor or very poor physical health | 1.00 |  |  |  |  |
| Average health | 0.81 | 0.66 | - | 1.01 | 0.058 |
| Good health | **1.30** | **1.09** | **-** | **1.55** | **0.003** |
| Very good health | 1.32 | 1.02 | - | 1.70 | 0.032 |
| Emotional health |  |  |  |  |  |
| Poor or very poor emotional health | 1.00 |  |  |  |  |
| Average emotional health | **0.65** | **0.52** | **-** | **0.82** | **0.000** |
| Good emotional health | **1.30** | **1.07** | **-** | **1.58** | **0.008** |
| Very good emotional health | 1.32 | 1.03 | - | 1.68 | 0.028 |
| FINANCIAL ACCESS |  |  |  |  |  |
| Has health insurance | **1.83** | **1.33** | **-** | **2.52** | **0.000** |
| Adequacy of insurance coverage |  |  |  |  |  |
| Does not suit my needs | 1.00 |  |  |  |  |
| Suits my needs quite well | **3.53** | **2.87** | **-** | **4.35** | **0.000** |
| Suits my needs very well | **3.37** | **2.08** | **-** | **5.46** | **0.000** |
| Income |  |  |  |  |  |
| Lowest | 1.00 |  |  |  |  |
| Second lowest | 1.09 | 0.84 | - | 1.41 | 0.530 |
| Middle | 1.11 | 0.85 | - | 1.44 | 0.451 |
| Second highest | 1.08 | 0.83 | - | 1.41 | 0.549 |
| Highest | 0.96 | 0.70 | - | 1.31 | 0.774 |
| IDEOLOGICAL BELIEFS |  |  |  |  |  |
| We should pay for own health care |  |  |  |  |  |
| Disagree | 1.00 |  |  |  |  |
| Somewhat agree | **1.54** | **1.27** | **-** | **1.88** | **0.000** |
| Strongly agree | **2.07** | **1.41** | **-** | **3.03** | **0.000** |
| *Extent of inequality in access* | **0.48** | **0.40** | **-** | **0.58** | **0.000** |

| UTILIZATION |  |  |  |  |  |
| --- | --- | --- | --- | --- | --- |
| N hospital visits over last year | **0.84** | **0.75** | **-** | **0.93** | **0.001** |
| N clinic visits over last year | 1.01 | 0.96 | - | 1.07 | 0.696 |
| MEDIA USE |  |  |  |  |  |
| *Uses social media for news* | **0.84** | **0.77** | **-** | **0.93** | **0.000** |
| Watches TV for news | 1.06 | 0.98 | - | 1.16 | 0.148 |
| PERCEPTIONS OF SERVICES |  |  |  |  |  |
| *Convenience of hospitals* | **1.25** | **1.07** | **-** | **1.46** | **0.005** |
| *Convenience of clinics* | **1.26** | **1.06** | **-** | **1.49** | **0.007** |
| *Clinics: value for money* | **1.56** | **1.34** | **-** | **1.82** | **0.000** |
| *Hospitals: value for money* | **1.67** | **1.42** | **-** | **1.96** | **0.000** |
| *Clinics: competence* | **1.60** | **1.30** | **-** | **1.98** | **0.000** |
| *Hospitals: competence* | **1.78** | **1.45** | **-** | **2.19** | **0.000** |
| *Likelihood of unethical practices* | **0.52** | **0.44** | **-** | **0.62** | **0.000** |
| **Bold: P<.01** |  |  |  |  |  |

| **Appendix S4. Supplementary analysis: social structure, employment and insurance type** | | | | | |
| --- | --- | --- | --- | --- | --- |
|  | Odds | 95% CI | | | p- |
|  | Ratio | lower | - | Upper | value |
| Male | 1.04 | 0.90 | - | 1.22 | 0.568 |
| Age 30-59 | 1.00 |  |  |  |  |
| Young (Age-18-29) | 0.91 | 0.68 | - | 1.21 | 0.504 |
| Old (Age 60+) | **1.40** | **1.12** | **-** | **1.75** | **0.004** |
| Education: Primary only | 1.00 |  |  |  |  |
| Junior high education | 1.00 | 0.76 | - | 1.30 | 0.973 |
| Senior high/technical | 0.70 | 0.53 | - | 0.94 | 0.017 |
| University | 0.75 | 0.53 | - | 1.07 | 0.118 |
| Rural residence | 1.53 | 1.07 | - | 2.19 | 0.021 |
| Non-agricultural residence registration | 0.95 | 0.70 | - | 1.31 | 0.770 |
| Local residence registration | 1.61 | 1.12 | - | 2.30 | 0.010 |
| Self-assessed physical health |  |  |  |  |  |
| Poor or very poor physical health | 1.00 |  |  |  |  |
| Average health | **1.62** | **1.18** | **-** | **2.24** | **0.003** |
| Good health | **2.29** | **1.75** | **-** | **3.00** | **0.000** |
| Very good health | **2.68** | **1.91** | **-** | **3.77** | **0.000** |
| Occupation: Manual labourer | 1.00 |  |  |  |  |
| Unemployed | 0.61 | 0.41 | - | 0.92 | 0.017 |
| Retired | 1.09 | 0.79 | - | 1.50 | 0.619 |
| Agricultural labourer | 1.21 | 0.91 | - | 1.60 | 0.185 |
| Professional | 0.77 | 0.54 | - | 1.11 | 0.162 |
| Clerical worker | 0.88 | 0.67 | - | 1.16 | 0.368 |
| Entrepreneur | 1.23 | 0.84 | - | 1.80 | 0.287 |
| Street vendor | 0.88 | 0.51 | - | 1.54 | 0.658 |
| Health professional | 0.94 | 0.55 | - | 1.62 | 0.833 |
| Type of employer: private sector | 1.00 |  |  |  |  |
| Public sector | 1.72 | 1.08 | - | 2.73 | 0.022 |
| State-owned enterprise | 0.92 | 0.68 | - | 1.26 | 0.615 |
| Self-employed | 1.09 | 0.82 | - | 1.45 | 0.551 |
| Income: Lowest | 1.00 |  |  |  |  |
| Second lowest | 1.30 | 0.95 | - | 1.77 | 0.097 |
| Middle | 1.37 | 1.00 | - | 1.87 | 0.047 |
| Second highest | 1.37 | 0.95 | - | 1.98 | 0.097 |
| Highest | 1.30 | 0.90 | - | 1.88 | 0.163 |
| Insured: Rural Cooperative Medical Scheme | 1.60 | 1.06 | - | 2.41 | 0.025 |
| Urban Employees Basic Medical Insurance | **1.46** | **1.11** | **-** | **1.91** | **0.006** |
| Urban Residents Basic Medical Insurance | 1.58 | 1.05 | - | 2.36 | 0.026 |
| Model fit statistics: -2LL fitted: 399,793, null: 484,743, difference: 84,950, df 30 (P<.001) | | | | | |
| **Bold: P<.01** |  |  |  |  |  |
